# Dr. Peter Broadt

**Published:** 1904-02

**Authors:** 


DR. PETER BROADT, a homeopathic physician of Buffalo, died January 21, 1904, aged 70 years.

